# Stability of Cell Wall Composition and Saccharification Efficiency in *Miscanthus* across Diverse Environments

**DOI:** 10.3389/fpls.2016.02004

**Published:** 2017-01-05

**Authors:** Tim van der Weijde, Oene Dolstra, Richard G. F. Visser, Luisa M. Trindade

**Affiliations:** ^1^Wageningen UR Plant Breeding, Wageningen University and ResearchWageningen, Netherlands; ^2^Graduate School Experimental Plant Sciences, Wageningen UniversityWageningen, Netherlands

**Keywords:** miscanthus, multi-location trial, genotype-by-environment interaction, stability, GGE biplot, biomass quality, ethanol, near-infrared spectroscopy (NIRS)

## Abstract

To investigate the potential effects of differences between growth locations on the cell wall composition and saccharification efficiency of the bioenergy crop miscanthus, a diverse set of 15 accessions were evaluated in six locations across Europe for the first 3 years following establishment. High-throughput quantification of cellulose, hemicellulose and lignin contents, as well as cellulose and hemicellulose conversion rates was achieved by combining near-infrared reflectance spectroscopy (NIRS) and biochemical analysis. Prediction models were developed and found to predict biomass quality characteristics with high accuracy. Location significantly affected biomass quality characteristics in all three cultivation years, but location-based differences decreased toward the third year as the plants reached maturity and the effect of location-dependent differences in the rate of establishment reduced. In all locations extensive variation in accession performance was observed for quality traits. The performance of the different accessions in the second and third cultivation year was strongly correlated, while accession performance in the first cultivation year did not correlate well with performance in later years. Significant genotype-by-environment (G × E) interactions were observed for most traits, revealing differences between accessions in environmental sensitivity. Stability analysis of accession performance for calculated ethanol yields suggested that selection for good and stable performance is a viable approach. Environmental influence on biomass quality is substantial and should be taken into account in order to match genotype, location and end-use of miscanthus as a lignocellulose feedstock.

## Introduction

To expedite the utilization of renewable plant biomass as an alternative to fossil fuel it is necessary to develop high yielding biomass crops producing biomass of high quality in different environments (van der Weijde et al., 2013). Several second-generation energy crops have potential as a lignocellulose feedstock for biofuel production, but one of the strongest contenders is miscanthus (Heaton et al., 2010). Miscanthus is a highly productive perennial grass with a high nutrient-use efficiency, owing to its highly efficient C4 photosynthesis system and ability to translocate minerals to underground rhizomes at the end of the cultivation year (Heaton et al., 2010). The genus *Miscanthus* comprises approximately 15 different species of which *M. sinensis*, *M. sacchariflorus* and their interspecific hybrids are considered to have the highest potential for biomass production (Jones and Walsh, 2001). These miscanthus species harbor great genetic diversity and occur naturally over a large geographical range in East Asia (Clifton-Brown et al., 2008). As a result miscanthus displays a wide adaptation to different soils types and climates, which may allow its exploitation as a second generation biofuel feedstock across a broad range of environments.

However, the potential of a lignocellulose feedstock for the production of biofuel is also highly determined by the compositional quality of the biomass. Lignocellulosic biomass is mainly composed of cellulose, hemicellulosic polysaccharides and lignin (Doblin et al., 2010). The content of polysaccharides determines how much fermentable sugars are theoretically available at a maximum conversion rate of 100%. The content of lignin, on the other hand, is one of the main factors that limit the extraction of fermentable sugars from the cell wall (Chundawat et al., 2011). Lignin is a complex aromatic polymer that crosslinks to hemicellulosic polysaccharides, forming a highly impermeable matrix that imparts strength to the plant cell wall and shields cell wall polysaccharides against chemical and enzymatic hydrolysis (Himmel and Picataggio, 2008; Chundawat et al., 2011). Cell wall compositional characteristics are therefore considered important quality criteria for biofuel feedstocks and the development of improved varieties with increased polysaccharide, reduced lignin content and increased saccharification efficiency is seen as crucial to reduce the production costs of cellulosic biofuels (Wyman, 2007; Torres et al., 2016; van der Weijde et al., 2017).

There is ample scope for the development of such varieties through breeding as extensive genetic variation for cell wall composition is found in miscanthus, with contents of cellulose ranging from ~26 to 51%, hemicellulosic polysaccharides from ~25 to 43% and lignin from ~5 to 15% of dry matter in senesced biomass (Allison et al., 2011; Qin et al., 2012; Zhao et al., 2014). Cell wall compositional characteristics, however, are complex polygenic traits and are commonly affected by environmental as well as genetic determinants. Cell wall biosynthesis, particularly lignin deposition, is spatially and temporally regulated during the development of the plant and like any other complex metabolic pathway it can be reprogrammed in response to environmental signals (Boerjan et al., 2003; Pauly and Keegstra, 2010). The effect of environment on miscanthus cell wall composition was first demonstrated by Hodgson and coworkers, who studied the extent of genotypic and environmentally derived variation in cell wall composition in a study at five field trial locations (Hodgson et al., 2010). They concluded that the degree of observed genotypic variation in cell wall composition indicated a high potential for breeding for biomass quality characteristics, but also stressed the significance of environmentally derived variation in cell wall composition. However, this study was only conducted for one growth year, while miscanthus is a perennial crop that exhibits considerable morphological and physiological changes following the first few years after establishment. The variation in miscanthus cell wall composition has never been examined across multiple locations and harvest years, nor is the effect this may have on biomass quality for biofuel production. Such information may reveal important insights into the stage at which genotype performance may accurately be assessed in breeding programs, as well as into the accuracy of single location vs. multi-locational trialing of germplasm.

In this study we investigated in-depth how differences between growth locations affect biomass quality in miscanthus. To this end we studied the cell wall composition and saccharification efficiency of a set of 15 accessions across different locations and cultivation years. The test comprised 4 *M. sacchariflorus*, 5 *M. sinensis* and 6 hybrid accessions, which were evaluated for 3 years in six locations across Europe: Aberysthwyth (United Kingdom, UK), Adana (Turkey, TR), Potash (Ukraine, UA), Moscow (Russia, RU), Stuttgart (Germany, DE) and Wageningen (Netherlands, NL). Our focus was on quality traits relevant to the production of bioethanol, but an increase in our understanding of cell wall composition in relation to genetic and environmental factors is relevant to many of the value-chains for which miscanthus biomass has potential. This is the first multi-year, multi-location study on biomass quality in miscanthus and these insights are highly relevant to the development of new varieties through breeding, as well as to the biorefinery industry, as we gain understanding of the compositional quality of miscanthus biomass grown across diverse environments.

## Materials and methods

### Plant materials

Fifteen miscanthus accessions, belonging to three different miscanthus species, were used in this study; five accessions of *M. sinensis*, including the commercial cultivar “*Goliath*,” four of *M. sacchariflorus*, including the commercial cultivar “*Robustus*,” and six hybrid accessions derived from crosses between *M. sinensis* and *M. sacchariflorus*, including the commercially-used clone “*M*. × *giganteus*” (Table 1). The accessions were tested in a multi-location trial with six locations (Table 2): Aberystwyth (UK), Adana (TR), Potash (UA), Moscow (RU), Stuttgart (DE), and Wageningen (NL). For a more detailed description of the trial sites, the reader is referred to Lewandowski et al. (2016). The trials were established using a completely randomized block design with three replications per accession between April and May 2012. The planting materials used to establish the trials were clones produced by *in vitro* propagation (OPM 1-11) or seed-derived plantlets (OPM 12-15). For each of the 15 accessions 49 plantlets were planted per plot in a 7-by-7 grid (total amount of plantlets = 6 locations × 15 accessions × 3 replicated plots × 49 plantlets per plot = 13.230). The planting density was two plants per m^2^, resulting in a plot size of 25 m^2^. Field trials were managed without irrigation, except for the trial in Adana, in which minimal irrigation was applied in the summer of the first year to ensure plant survival. All trials were fertilized once, prior to establishment of the trials, with a single application of 44 kg P ha^−1^ and 110 kg K ha^−1^. The trials were harvested between January and April for three consecutive years after establishment of the trials (first harvest 2013, second harvest 2014, third harvest 2015). To minimize potential border effects, for each plot only the inner nine plants (3-by-3 grid) were harvested (the two outer rows of plants of every plot being regarded as border plants), bundled and processed further. Each bundle of biomass was weighed and subsequently a ~400 gram subsample from every bundle was drawn randomly for determination of moisture content. Moisture content was determined after chopping and drying of the subsample in a forced-air oven at 60°C for 72 h and used for the calculation of dry matter yields per plot. A second ~400 gram subsample of shoots was randomly drawn from each bundle and stripped from leaves. The remaining stem material was chopped and dried in a forced-air oven at 60°C for 72 h and used for the calculation of stem dry matter yields per plot. Subsequently, the dried stem material was ground using a hammer mill with a 1-mm screen and used for biomass quality analyses [*n* = 810 (3 years × 6 locations × 15 accessions × 3 blocks)].

**Table 1:** **Accession, species and propagation information of the 15 *miscanthus* accessions used in this study**.

**Accession**	**Species**	**Plants**
OPM 1	*M. sacchariflorus*	*In vitro*
OPM 2	*M. sacchariflorus*	*In vitro*
OPM 3	*M. sacchariflorus*	*In vitro*
OPM 4	*M. sacchariflorus “Robustus”*	*In vitro*
OPM 5	Hybrid	*In vitro*
OPM 6	Hybrid	*In vitro*
OPM 7	Hybrid	*In vitro*
OPM 8	Hybrid	*In vitro*
OPM 9	Hybrid “*M*. × *giganteus*”	*In vitro*
OPM 10	*M. sinensis*	*In vitro*
OPM 11	*M. sinensis “Goliath”*	*In vitro*
OPM 12	*M. sinensis*	Seed
OPM 13	*M. sinensis*	Seed
OPM 14	*M. sinensis*	Seed
OPM 15	Hybrid	Seed

**Table 2:** **Location characteristics and long term annual and growth season (approximated April–September) temperature and rainfall for the six trial locations**.

**Location name**	**Latitude**	**Longitude**	**Altitude (m)**	Air Temperature^*^, °C		Rainfall^*^, mm	
				**Annual**	**April to Sept**	**Annual**	**April to Sept**
Aberystwyth (UK)	52.43	−4.01	39	9.7	13.8	1038	401
Adana (TR)	37	35	27	19.0	26.1	575	75
Moscow (RU)	55	37	140	4.1	14.8	644	347
Potash (UA)	48.89	30.44	237	8.9	18.5	537	300
Stuttgart (DE)	48.74	8.93	463	9.8	16.4	725	379
Wageningen (NL)	51.59	5.39	10	10.3	15.8	826	376

* *Climate data for Adana, 2000–2011; for Stuttgart, 1988–1999; for Potash, 2003–2012; for Wageningen, 2002–2012; for Aberystwyth, 1954–2000, and for Moscow, 1881–1980*.

### Fiber analyses

Neutral detergent fiber (NDF), acid detergent fiber (ADF) and acid-detergent lignin contents (ADL) of stem dry matter were determined according to protocols developed by Ankom Technology (ANKOM Technology Corporation, Fairpoint, NY), which are essentially based on the work of Goering and Van Soest (Van Soest, 1967; Goering and Van Soest, 1970). NDF and ADF fractions are the residues remaining after refluxing the samples in neutral or acid detergent solutions, respectively, using an ANKOM 2000 Fiber Analyzer (ANKOM Technology Corporation, Fairpoint, NY). Acid detergent lignin was determined after 3-h hydrolysis of the ADF residue in 72% H_2_SO_4_ with continuous shaking. All analyses were performed in triplicate and fiber fractions were expressed in gram per kg dry matter.

### Determination of saccharification efficiency

Saccharification efficiency of the samples was assessed by the conversion of cellulose into glucose and hemicelluloses into xylose using a mild alkaline pretreatment and enzymatic saccharification reaction, essentially as described by van der Weijde et al. (2016a). Reactions were carried out in triplicate using 500 mg subsamples per stem sample. All subsamples were incubated for 13 min with α-amylase (thermostable α-amylase, ANKOM Technology Corporation, Fairpoint, NY), followed by three 5 minincubations with warm deionized water (~60°C) in order to remove interfering soluble sugars. The remaining biomass was then subjected to a mild alkaline pretreatment, carried out in 50 ml plastic centrifuge tubes with 15 ml 2% NaOH at 50°C with constant shaking (160 RPM) for 2 h in an incubator shaker (Innova 42, New Brunswick Scientific, Enfield, CT). In this study the objective of the pretreatment was not to maximize cellulose conversion but to treat samples to better allow discrimination of genotypic differences in cellulose conversion efficiency. Pretreated samples were washed to neutral pH with deionized water (2 ×, 5 min, 50°C) and with 0.1 M sodium citrate buffer (pH 4.6, 5 min, 50°C).

Saccharification reactions were subsequently carried out according to the NREL Laboratory Analytical Procedure “Enzymatic saccharification of lignocellulosic biomass” (Selig et al., 1996). Pretreated samples were hydrolyzed for 48 h with 300 μl (25.80 mg of enzyme) of the commercial enzyme cocktail Accellerase 1500 (DuPont Industrial Biosciences, Leiden, NL) supplemented with 15 μl (0.12 mg of enzyme) endo-1,4-β-xylanase M1 (EC 3.2.1.8, Megazyme International Ireland, Bray, IE) in an incubator shaker (Innova 42, New Brunswick Scientific, Enfield, CT) set at 50°C and constant shaking (160 RPM). This enzyme mixture has the following reported specific activities: endoglucanase 2200–2800 carboxymethylcellulose (CMC) Units per gram, beta-glucosidase 450–775 p-nitrophenol-beta-D-glucoside (pNPG) Units per gram and the xylanase has an endoxylanase activity of 230 Units per mg. Reactions were carried out in 44 ml 0.1 M sodium citrate buffer (pH 4.6), containing 1.3 ml of a 1% benzoate solution for the prevention of microbial contamination.

Glucose and xylose contents in the enzymatic saccharification liquors were determined using enzyme-linked D-glucose (R-Biopharm, Darmstadt, DE) and D-xylose (Megazyme International Ireland, Bray, IE) assay kits. These assays were adapted to a 96-well microplate format and the increases in sample absorption following enzyme-mediated conversion reactions were spectrophotometrically determined at 340 nm using a Bio-Rad Microplate Reader (Bio-Rad, Richmond, CA, USA). Spectrophotometric determination of each sample was done in duplicate and all absorbance measurements were corrected using blanks, containing demineralized water instead of sample solution. Glucose and xylose release was determined by calculating the glucose and xylose content, respectively, in the saccharification liquor from absorbance measurements using Equation (1).

(1) $$\begin{array}{r} Glucose/xylose\;release\;\left(mg\right)=\frac{V\;\times \;MW}{\varepsilon \;\times \;d\;\times \;v\;\times \;1000}\\ \times \;df\times \Delta Abs \end{array}$$

where *V* = final well volume (3.02 ml for glucose and 2.97 ml for xylose measurement); *MW* = molecular weight of glucose (180.16 g/mol for glucose and 150.13 for xylose); ε = the molar extinction coefficient of NADPH or NADH for glucose and xylose measurements, respectively (6.3 L × mol^−1^ × cm^−1^); *d* = light path-length (=1.016 cm); *v* = sample volume (0.1 ml); *df* = dilution factor (10 for glucose and 5 for xylose measurement); and Δ*Abs* = increase in sample absorbance, corrected for the increase in blank absorbance. Cellulose conversion (CelCon, %) and hemicellulose conversion (HemCon, %) rates were calculated from the release of glucose/xylose relative to the content of cellulose/hemicellulose, respectively, as detailed in Equations (2) and (3).

(2) $$\begin{array}{r} \text{CelCon }\%=\frac{\text{Glucose release }\left(\text{mg}\right)}{\text{CEL}\times 1.111\times \text{S}}\times 100\% \end{array}$$

(3) $$\begin{array}{r} \text{HemCon }\%=\frac{\text{Xylose release }\left(\text{mg}\right)}{\text{HEM}\times 1.136\times \text{S}}\times 100\% \end{array}$$

where CEL = cellulose content (in g / kg dm = mg / g dm) in the sample, calculated as described below; *1.111* = the mass conversion factor that converts cellulose to equivalent glucose (the molecular weight ratio of 180.16–162.16 g/mol for glucose and anhydro-glucose) (Dien, 2010); HEM = hemicellulose content (in g/kg dm = mg/g dm) in the sample, calculated as described below; 1.136 = the mass conversion factor that converts xylan to equivalent xylose (the molecular weight ratio of 150.13–132.12 g/mol for xylose and anhydro-xylose) (Dien, 2010); and S = the amount of sample material in gram dry matter. Calculated ethanol yield (CEY, g / kg dm) was calculated by considering full conversion of all the released glucose and xylose into ethanol, as detailed in equation 4.

(4) $$\begin{array}{r} \text{CEY }\left(\text{g}/\text{kg dm}\right)=\frac{\text{Glucose release }\left(\text{mg}\right)\times 2\times \text{MwE}}{\text{S }\times \text{ MwG}}\\ +\;\frac{\text{Xylose release }\left(\text{mg}\right)\times \frac{3}{5}\times \text{MwE}}{\text{S}\times \text{MwX}} \end{array}$$

where MwE = molecular weight of ethanol (= 46.06844 g/mol); MwG = molecular weight of glucose (180.15588 g/mol); MwX = molecular weight of xylose (= 150.13 g/mol); S = the amount of sample material in gram dry matter; multiplication factors 2 and $\frac{3}{5}$ refer to the amount of ethanol molecules formed from one molecule of glucose and xylose, respectively.

### Analysis of miscanthus biomass using near infrared spectroscopy (NIRS)

Multivariate prediction models based on near-infrared (NIR) spectral data were developed to allow high-throughput prediction of biomass quality traits. Near-infrared absorbance spectra of stem and leaf samples were obtained using a Foss DS2500 near-infrared spectrometer (Foss, Hillerød, Denmark). Averaged spectra were obtained consisting of 8 consecutive scans from 400 to 2500 nm using an interval of 2 nm using ISI-Scan software (Foss, Hillerød, Denmark). Obtained spectra were further processed by weighted multiplicative scatter correction and mathematical derivatization and smoothing treatments using WinISI 4.9 statistical software (Foss, Hillerød, Denmark). These statistical transformations of spectra help to minimize effects resulting from light scatter and differences in particle size. Parameters for derivatization and smoothing were set at 2-6-4-1, in which the first number of this mathematical procedure refers to order of derivatization, the second number to the gap in the data-points over which the derivation is applied and the third and fourth number refers to the number of data-points used in the smoothing of the first and second derivative.

For the creation of prediction models a calibration set of 250 samples was selected from the complete set of samples (*n* = 810): 110 samples of the first cultivation year, 80 samples of the second cultivation year and 60 samples of the third cultivation year, all selected at random or for being identified by the software as spectral outliers. The biochemical reference data and near-infrared spectra of the calibration samples were used for the development and cross-validation of prediction models using WinISI version 4.9 (Foss, Hillerød, Denmark). The prediction equations were generated using modified partial least squares regression analyses (Shenk and Westerhaus, 1991). The optimal number of principal components used for development of the prediction models was manually determined to be 8. Inclusion of more factors hardly improved the prediction models as determined by validation and increases the risk of “over-fitting” of the data. The prediction models were validated using the squared Pearson coefficient of correlation (*r*^2^) between predicted and biochemical data and by evaluating for these samples the standard error of cross-validation (SECV) for each of the traits (Table 3). As good correlations (*r* > 0.82) were found between predicted and biochemical data, and the results of cross-validation were satisfactory, the prediction models were subsequently used to determine NDF, ADF, ADL, cellulose conversion, and hemicellulose conversion for all 810 stem samples. The predicted fiber fractions were used to calculate the concentrations (in g/kg dm) of cell wall (NDF) cellulose (CEL, equals ADF - ADL), hemicellulosic polysaccharides (HEM, equals NDF - ADF) and acid-detergent lignin (LIG, equals ADL) in stem dry matter.

**Table 3:** **Summary of cross-validation statistics of mPLS models used for the prediction of biomass quality traits from NIRS spectral data**.

**Constituent**	Samples^*^	**Chemical analysis**			**NIRS prediction**			***r*****^2^^¥^**	**SECV^§^**
		**Mean**	**Min**	**Max**	**Mean**	**Min**	**Max**		
NDF (g/kg dm)	246	85.04	71.55	92.69	85.04	71.28	92.35	0.99	0.88
ADF (g/kg dm)	243	54.96	38.43	68.55	54.97	39.40	68.47	0.99	1.13
ADL (g/kg dm)	239	9.22	4.88	14.45	9.20	5.26	14.42	0.88	0.79
Cellulose conversion (%)	237	29.89	8.17	52.10	30.21	13.14	46.81	0.92	3.22
Hemicellulose conversion (%)	243	12.43	5.84	22.20	12.34	6.70	20.27	0.82	2.06

* , Sample number varies as for every trait different samples may be removed by the software as outliers; depending on the model

¥ r^2^, coefficient of determination;

§ *SECV, Standard error of cross-validation*.

### Statistical analyses

General analyses of variance (ANOVA) were performed to determine the significance of accession differences, locations, cultivation years and their interactions (*p* < 0.05) on cell wall composition and saccharification efficiency. Variance analyses were performed following the standard procedure of a mixed effect model with a random genetic effect, a fixed location effect, a random year effect and a fixed block effect, following the model (5):

(5) $$\begin{array}{r} R_{ijkr}=\mu +G_{i}+L_{j}+Y_{k}+B_{r}\;\left(L_{j}Y_{k}\right)+GL_{ij}+GY_{ik}\\ +\;LY_{jk}+GLY_{ijk}+e_{ijkr} \end{array}$$

where *R*_*ijkr*_ is the response variable, μ is the grand mean, *G*_*i*_ is the genotype effect, *L*_*j*_ is the location effect, *Y*_*k*_ is the year effect, *B*_*r*_ *(L*_*j*_ *Y*_*k*_*)* is the block effect, *GL*_*ij*_ is the genotype-by-location interaction, *GY*_*ik*_ is the genotype-by-year interaction, *LY*_*jk*_ is the location-by-year interaction, *GLY*_*ijk*_ is the genotype-by-location-by year interaction and *e*_*ijkr*_ is the residual error. To study the potential of early selection correlation analyses were performed on accession means to identify the significance (*p* < 0.05) of correlations between traits across cultivation years using Pearson's correlation coefficients. In addition a Finlay Wilkinson stability analysis was performed using the calculated ethanol yield data of the third cultivation year (6) (Finlay and Wilkinson, 1963; Malosetti et al., 2013):

(6) $$\begin{array}{r} R_{ij}=\mu +G_{i}+\beta_{i}\times L_{j}+e_{ij} \end{array}$$

where *R*_*ij*_ is the response variable, μ is the grand mean, *G*_*i*_ is the genotype effect, ß_*i*_ is the regression coefficient of accession *i* for environment *j* (environmental sensitivity), *L*_*j*_ is a measure of environmental quality determined by the mean performance of accessions for CEY in environment *j* and *e*_*ij*_ is the residual error. Accession means per location for the third cultivation year were also used to fit a GGE model by singular value decomposition of environment-centered genotype by location data (7) (Malosetti et al., 2013):

(7) $$R_{ij}=\mu +L_{j}+\sum_{k\;=\;1}^{k}{ß}_{ik}\times L_{jk}+e_{ij}$$

where accession performance is explained by K multiplicative terms (k = 1…K), each formed by the product of environmental sensitivity (ß_*ik*_) of accession *i* and environmental score (*L*_*jk*_). A GGE biplot was constructed in which accession performance (accounting for both genotype main effect and genotype-by-location interaction) across environments is visualized in a scatter plot of accession and location scores for the first two principal components (Yan and Kang, 2002; Malosetti et al., 2013). Correlation analyses were performed to identify the significance, strength and direction of interrelationships between morphological and quality traits using Pearson's correlation coefficients. All statistical analyses were performed using Genstat for Windows, 18th edition software package (VSN International, Hemel Hempstead, UK).

## Results and discussion

### Impact of accession, location, and cultivation year on biomass quality

Cell wall composition and saccharification efficiency of 15 miscanthus accessions were studied in a multi-year, multi-location field experiment. Analyses of variance revealed that cell wall composition and saccharification efficiency differed significantly between accessions and that these traits were strongly affected by both trial location and cultivation year (Tables 4, 5). Miscanthus is a perennial crop that typically matures in 2–5 years, depending on the environmental conditions. During this process of maturation, miscanthus shows a pattern of increasing yields during the establishment phase, until at full maturity a plateau phase is reached, with relatively stable yields (Christian and Haase, 2001; Christian et al., 2008; Gauder et al., 2012; Hulle et al., 2012; Arnoult et al., 2015). Here we show that during this establishment phase, cell wall composition is changing as the crop matures.

**Table 4:** **Analyses of variance for cell wall composition of 15 miscanthus accessions grown in six locations and evaluated for three successive cultivation years (2012–2013, 2013–2014 and 2014–2015)**.

Source of variation^*^	**Degrees of freedom**	**NDF (g/kg dm)**		**CEL (g/kg dm)**		**HEM (g/kg dm)**		**LIG (g/kg dm)**	
		***Mean squares***	***F prob.***	***Mean squares***	***F prob.***	***Mean squares***	***F prob.***	***Mean squares***	***F prob.***
L	5	104619.6	<0.0001	145509.5	<0.0001	22834.8	<0.0001	8375.0	<0.0001
Residual^a^	12	489.8		835.1		992.9		196.1	
G	14	9644.3	<0.0001	18230.8	<0.0001	28602.2	<0.0001	5027.7	<0.0001
Y	2	150768.8	<0.0001	309417.8	<0.0001	84714	<0.0001	13962.3	<0.0001
GL	70	1308.7	0.0002	1312.6	<0.0001	697.5	<0.0001	143.1	0.0904
GY	28	960.2	0.0632	1139.5	0.0059	1548.2	<0.0001	465.4	<0.0001
LY	10	37187.4	<0.0001	31550	<0.0001	6469.7	<0.0001	2283.3	<0.0001
GLY	138	637.2	<0.0001	579.9	<0.0001	297.5	0.000	109.2	<0.0001
Residual^b^	500	242.8		308		184.6		50.3	

* G, Genotype; L, Location; Y, Year; GL, Genotype-by-location interaction; GY, Genotype-by-year interaction; LY, Location-by-year interaction; GLY, Genotype-by-location-by-year interaction.

a Residual, Residual block stratum;

b Residual, Residual block^*^units stratum.

**Table 5:** **Analyses of variance for conversion efficiency and calculated ethanol yield (CEY) of 15 miscanthus accessions grown in six locations and evaluated for three successive cultivation years (2012–2013, 2013–2014, and 2014–2015)**.

Source of variation^*^	**Degrees of freedom**	**CelCon (%)**		**HemCon (%)**		**CEY (g/kg dm)**	
		***Mean squares***	***F prob.***	***Mean squares***	***F prob.***	***Mean squares***	***F prob.***
L	5	2071.2	<0.0001	184.6	<0.0001	3171.3	<0.0001
Residual^a^	12	23.8		2.5		84.2	
G	14	283.2	<0.0001	51.1	<0.0001	3171.3	<0.0001
Y	2	18801.3	<0.0001	1151.8	<0.0001	84.2	<0.0001
GL	70	21.1	0.0003	3.1	0.0639	141.1	0.0099
GY	28	26.2	0.0002	2.5	0.3834	205.2	0.0007
LY	10	508.0	<0.0001	46.1	<0.0001	2836.9	<0.0001
GLY	138	10.7	<0.0001	2.3	<0.0001	88.4	<0.0001
Residual^b^	500	4.8		1.1		25.1	

* G, Genotype, L, Location, Y, Year; GL, Genotype-by-location interaction; GY, Genotype-by-year interaction; LY, Location-by-year interaction; GLY, Genotype-by-location-by-year interaction.

a Residual, Residual block stratum;

b Residual, Residual block^*^units stratum.

**Figure 1:**
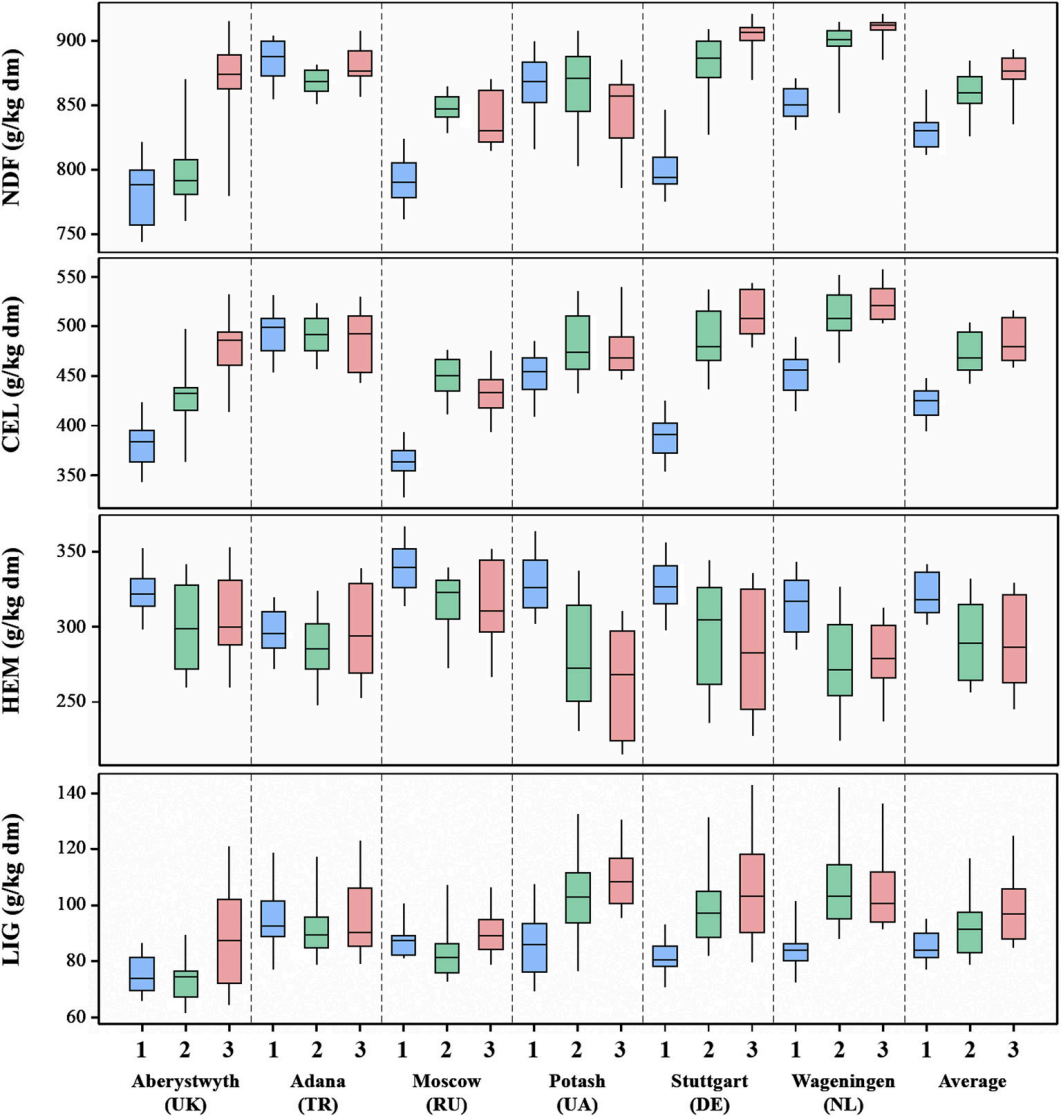
**Variation in accession means of 15 miscanthus accessions for cell wall composition characteristics in six growth locations and three cultivation years (1 = 2012–2013, 2 = 2013–2014, and 3 = 2014–2015)**.

**Figure 2:**
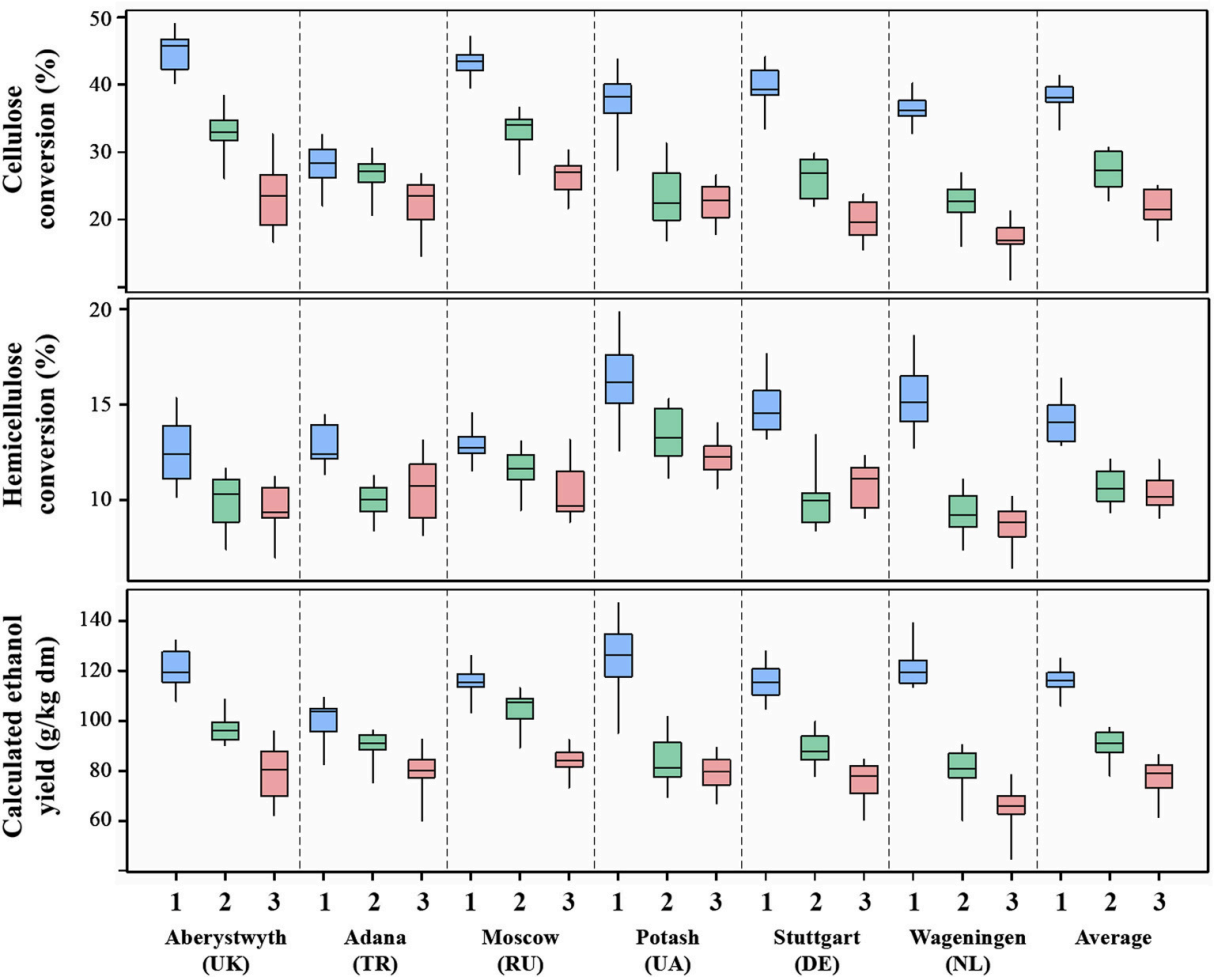
**Variation in accession means of 15 miscanthus accessions for conversion efficiency characteristics in six growth locations and three cultivation years (1 = 2012–2013, 2 = 2013–2014, and 3 = 2014–2015)**.

Boxplots of biomass quality traits are provided in Figures 1, 2, that depict the average and range in the performance of 15 accessions for each of the locations and cultivation years. Biomass composition in the first cultivation year differed considerably from that in the second and third, with substantially lower overall cell wall (NDF), cellulose (CEL) and to some extend lignin (LIG) contents and substantially higher contents of hemicellulosic polysaccharides (HEM) in the first year. For cultivation years 1, 2, and 3 mean NDF contents were ~829, ~860, and ~876 g/kg dm, respectively. Similarly, mean CEL contents were ~422, ~474, and ~485 g/kg dm and LIG contents were ~85, ~93, and ~99 g/kg dm, respectively. Mean HEM contents decreased from ~322 in the first, to ~293 in the second and ~291 g/kg dm in the third year (Figure 1). Saccharification efficiency also differed substantially between cultivation years (Table 5) and was much higher in the first year than in the second or third year (Figure 2). Mean cellulose conversion (CelCon) reduced from ~38% in the first year to ~27% in the second and ~22% in the third year. Similarly, mean hemicellulose conversion (HemCon) reduced from ~14% in the first, to ~11 in the second and ~10% in the third year. These changes in biomass composition and quality culminated in substantial reductions in mean calculated ethanol yields (CEY) from ~117 in the first, to 91 in the second and 77 g/kg dm in the third cultivation year (Figure 2). The ethanol yields reported in this study are relatively low compared to industrial standards, because very mild pretreatment conditions were chosen in this study as these are better suited to expose genotypic differences in saccharification efficiency (Torres et al., 2013; van der Weijde et al., 2017).

Biomass quality traits were also highly influenced by the different environments in the trial location (Tables 4, 5). Extreme differences came to light between Adana and the other locations for NDF, CEL, CelCon, and CEY. These differences were particularly evident in the first harvest year (Figures 1, 2), which may be attributed to location-dependent differences in the rate of establishment, although inter-annual variation in weather conditions may also have contributed. Miscanthus has a tendency to mature more slowly at northern latitudes than at latitudes closer to the equator (Lewandowski et al., 2000; Clifton-Brown et al., 2001). After the first growth season miscanthus stands in Adana already reached near plateau yields (on average 8 t dm ha^−1^), while yields in the other locations did not reach above 2 t dm ha^−1^ (Kalinina et al., unpublished data). However, these differences will become less pronounced toward the third harvest year, as stands in all locations start to reach full maturity.

For more in-depth evaluation of location differences in biomass quality, the material from the third cultivation year - assumed to represent mature, well-established miscanthus stands in all locations - was further examined (Table 6). Biomass composition varied extensively across locations, with mean NDF content ranging from 840 to 910 g/kg dm, CEL content from 434 to 524 g/kg dm, HEM content from 262 to 316 g/kg dm and LIG content from 89 to 109 g/kg dm (Table 6). The highest NDF and CEL contents were observed in Wageningen, while the lowest were observed in Moscow. These two locations were found to be the most contrasting of the evaluated locations regarding cell wall composition. Locations also differed extensively in saccharification efficiency. Mean cellulose conversion ranged from 17.3 to 26.4% across locations, with the lowest rate observed in Wageningen and the highest in Moscow. Likewise, mean hemicellulose conversion ranged from 8.7 to 12.3%, with the lowest rate observed in Wageningen and the highest in Potash. Calculated ethanol yields ranged from 65.6 to 83.5 g/kg dm across locations, with the highest yields for Moscow and the lowest for Wageningen. Which environmental parameters underlie such location-based differences in cell wall composition needs to be further investigated using a wider range of environments. However, variations in cell wall composition and cellulose degradation efficiency of natural miscanthus ecotypes in China were associated to latitude and total annual sunshine hours of the original habitat (Zhao et al., 2014). Furthermore, drought stress was recently identified as an environmental factor with implications for cell wall composition, increasing both cellulose content and saccharification efficiency of miscanthus (van der Weijde et al., 2016b).

**Table 6:** **Summary table of average, range and least significant differences for biomass quality traits of 15 accessions evaluated in six locations (cultivation year 3, 2014–2015)**.

**Trait**	**Statistic**	**Aberystwyth (UK)**	**Adana (TR)**	**Moscow (RU)**	**Potash (UA)**	**Stuttgart (DE)**	**Wageningen (NL)**	**Mean**	**Range**	**LSD**
**LOCATION**										
NDF	**Average**	**871.8**	**881.0**	**839.5**	**847.1**	**904.3**	**909.9**	875.6	70.4	6.5
(g/kg dm)	Range	135.1	49.9	54.8	98.8	50.7	34.9	70.7		
	CV_t_ (%)^§^	3.7	1.5	2.5	3.6	1.3	0.9	2.3		
	LSD^¥^	40.9	32.8	18.0	24.8	10.9	9.0			
Cellulose	**Average**	**478.1**	**487.3**	**433.7**	**476.1**	**513.2**	**524.4**	485.5	90.7	7.3
(g/kg dm)	Range	117.4	86.7	81.5	92.4	64.3	53.4	82.6		
	CV_t_ (%)	6.4	6.3	6.0	5.8	4.5	3.4	5.4		
	LSD	43.7	34.7	19.2	24.9	16.3	19.4			
Hemicellulose	**Average**	**305.3**	**298.6**	**315.6**	**262.3**	**284.1**	**280.7**	291.1	53.3	5.5
(g/kg dm)	Range	93.0	85.3	84.0	94.7	107.7	74.9	89.9		
	CV_t_ (%)	8.8	10.5	8.8	13.5	13.8	8.7	10.7		
	LSD	27.4	19.9	18.0	15.0	17.3	21.9			
Lignin	**Average**	**88.5**	**95.0**	**90.2**	**108.8**	**107.0**	**104.8**	99.0	20.3	3.1
(g/kg dm)	Range	56.0	43.4	27.3	34.7	63.1	44.6	44.9		
	CV_t_ (%)	18.3	13.2	9.2	9.7	18.4	12.2	13.5		
	LSD	19.8	9.7	6.8	7.2	11.6	11.4			
Cellulose	**Average**	**23.3**	**22.5**	**26.4**	**22.7**	**20.0**	**17.3**	22.0	9.0	0.9
conversion	Range	16.2	12.3	8.7	8.9	8.3	10.4	10.8		
(%)	CV_t_ (%)	18.3	15.0	10.1	12.3	14.0	14.1	14.2		
	LSD	19.8	3.6	2.0	3.0	2.0	2.2			
Hemicellulose	**Average**	**9.6**	**10.5**	**10.4**	**12.3**	**10.8**	**8.7**	10.4	3.6	0.4
conversion	Range	4.3	5.0	4.3	3.5	3.3	3.8	4.0		
(%)	CV_t_ (%)	11.9	14.8	12.7	8.0	10.3	11.0	11.5		
	LSD	2.2	1.7	1.6	1.6	2.0	1.4			
CEY^*^	**Average**	**79.3**	**79.8**	**83.5**	**79.5**	**75.5**	**65.6**	77.2	17.9	2.0
(g/kg dm)	Range	34.0	32.5	19.3	22.5	24.7	34.2	27.9		
	CV_t_ (%)	12.5	9.8	6.3	8.7	9.7	12.3	9.9		
	LSD	13.4	8.3	4.9	7.7	6.5	6.1			

* CEY, Calculated ethanol yield;

§ CV_t_, Coefficient of trait variation (standard deviation over genotype means/location mean × 100%);

¥ *LSD, least-significant difference (0.05)*.

Despite the large effects of location and cultivation year, significant variation in genotype performance was also evident (Tables 4, 5). As can be seen in Table 6, the range of variation among accession within each location was extensive. Mean CEY over all locations was 77.2 g/kg dm with a mean range in variation among accessions of 27.9 g/kg dm (Table 6). To exemplify the extent of variation in accession performance we zoom in on the performance of accessions OPM-9 and OPM-13 in the third harvest year. Averaged across all locations, OPM-9 was shown to have a much higher mean lignin content (125 g/kg dm) then OPM-13 (85 g/kg dm, Table 7). This difference in lignin content and other cell wall characteristics contributed to the much higher CEY for OPM-13 (83 g/kg dm) compared to OPM-9 (61 g/kg dm). It was previously shown that OPM-9 (*M*. × *giganteus*), the most widely exploited miscanthus variety, has a considerably lower quality for biofuel production compared to many other accessions (van der Weijde et al., 2016a), which is shown here to be the case across diverse environments.

**Table 7:** **Mean and variation in accession performance of 15 *Miscanthus* accessions over six trial locations (cultivation year 3, 2014–2015)**.

**Accession**	**NDF (g/kg dm)**		**CEL (g/kg dm)**		**HEM (g/kg dm)**		**LIG (g/kg dm)**		**CelCon %**		**HemCon %**		**CEY (g/kg dm)**	
	**Mean**	**Range**	**Mean**	**Range**	**Mean**	**Range**	**Mean**	**Range**	**Mean**	**Range**	**Mean**	**Range**	**Mean**	**Range**
OPM-1	893.0	47.0	516.1	63.5	259.2	75.1	117.7	30.7	19.6	6.0	10.7	3.5	73.0	15.0
OPM-2	835.6	106.0	470.2	96.1	269.4	83.6	96.1	51.8	25.1	12.1	11.5	2.6	84.1	19.9
OPM-3	878.5	103.3	511.1	109.5	252.3	60.9	115.1	38.1	19.3	8.7	11.1	5.3	71.5	19.0
OPM-4	876.7	92.4	509.9	87.4	260.5	81.2	106.4	32.2	21.5	9.6	11.9	4.3	79.5	24.0
OPM-5	892.7	67.2	512.0	66.1	282.5	48.0	98.1	23.1	19.9	6.4	10.8	3.7	75.2	16.7
OPM-6	859.8	80.9	478.9	81.0	286.6	37.3	94.3	12.8	24.6	6.5	12.1	4.4	86.6	13.9
OPM-7	888.9	46.2	479.6	67.4	311.3	65.0	98.0	19.1	20.2	8.4	9.2	5.2	71.1	21.7
OPM-8	876.9	89.5	480.8	100.5	291.7	32.2	104.4	20.2	20.8	10.4	10.1	2.8	73.1	18.7
OPM-9	869.3	120.3	499.1	91.4	245.6	41.4	124.7	36.6	16.8	11.4	10.1	4.2	61.3	29.1
OPM-10	889.0	76.8	505.5	109.9	286.4	61.7	97.2	20.1	20.5	12.4	10.6	4.3	75.4	27.7
OPM-11	878.7	54.0	461.2	83.1	329.2	51.5	88.3	28.5	23.7	8.8	9.2	4.6	79.2	16.7
OPM-12	873.0	88.2	463.7	127.1	322.5	53.7	86.8	24.4	24.4	11.3	9.0	2.6	80.0	20.6
OPM-13	873.1	86.3	459.2	107.8	328.7	45.0	85.1	24.6	25.2	10.9	9.7	2.8	83.3	17.0
OPM-14	880.1	61.2	472.2	91.2	322.5	36.9	85.4	27.3	24.2	8.3	9.7	3.2	82.5	17.7
OPM-15	868.7	86.3	462.4	107.6	318.3	68.0	88.0	24.4	24.8	11.4	9.8	3.3	82.2	19.8

The extent of variation amongst accessions in cell wall composition and conversion efficiency was not equal across locations (Table 6). In the third harvest year, the coefficient of trait variation (CV_*t*_) across locations ranged from 0.9 to 3.7% for NDF, 3.4–6.4% for cellulose, 8.7–13.5% for hemicellulosic polysaccharides and 9.2–18.4% for lignin (Table 6). This showed that across locations particularly large variation in accession performance was observed for hemicelluloses and lignin. Variation in accession performance for conversion rates was also unequal across locations, with CV_*t*_ ranging from 10.1 to 18.3% for cellulose conversion and 8.0–14.8% for hemicellulose conversion. For four out of seven evaluated traits the largest variation in accession performance in the third year was observed in Aberystwyth.

### Stability of accession performance

**Figure 3:**
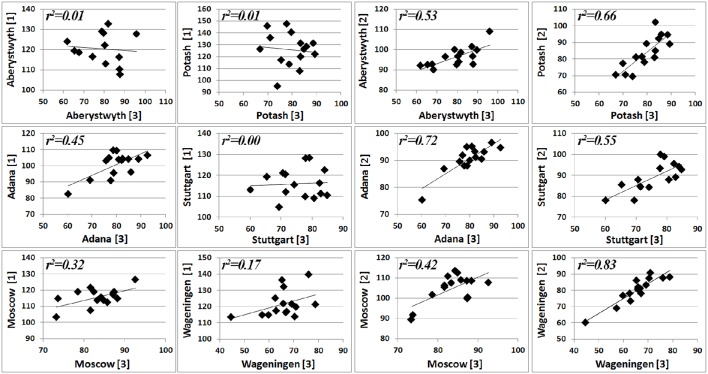
**Scatter plot matrix of calculated ethanol yields (g/kg dm) of the first [1] and the second [2] cultivation year of 15 miscanthus accessions in six locations compared to that of the third cultivation year [3]**.

We observed that miscanthus cell wall composition is not stable during the establishment phase of miscanthus. Moreover, variation in accession performance differed across cultivation year, as indicated by the significance of genotype-by-year interaction effects (Tables 4, 5). Therefore, early prediction of genotype performance may not be reliable. For each location, correlations of accession performance for calculated ethanol yield across the different harvest years are depicted in Figure 3. A low similarity (*r*^2^ < 0.32) in accession performance between the first and the third cultivation year was observed for all locations except for Adana (*r*^2^ = 0.45). However, for all locations accession performance in CEY in the second cultivation year correlated reasonably well with that in the third cultivation year (*r*^2^ = 0.42–0.83). Previously, Arnoult et al. (2015), already indicated that biomass quality in miscanthus harvested in the third cultivation year was reliably representative of that in the fourth and the fifth year in a single location. Here we validate that conclusion using data from multiple environments and even support that performance at full maturity can be estimated with reasonable accuracy from accession performance after two cultivation years. In contrast, selection for CEY based on CEY values obtained after 1 year of cultivation is not recommended, due to its low predictive value of performance at full maturity.

The results also showed that some accessions performed more stable across the different environments than others and that ranking of accessions differs across locations. Such differential ranking was observed for all evaluated traits, except for lignin and hemicellulose conversion efficiency, as indicated by the statistical significance of genotype-by-environment interactions (Tables 4, 5). When variance was analyzed on data of the third cultivation year only, statistically significant genotype-by-environment interactions were observed for all traits (Supplementary Tables S1, S2). This is the first report on genotype-by-location interactions for cell wall components and saccharification efficiency in miscanthus. Such interactions may have important implications for the set-up of selection experiments, as they implicate that the relative ranking of accessions is dependent on the environment. Dealing with large genotype-by-environment interaction in breeding programs usually means that germplasm has to be trialed in multiple locations as selection based upon data from a single experiment might lead to wrong selection decisions. However, like for several forage crops such as silage maize (Dolstra et al., 1992; Cox et al., 1994; Argillier et al., 1997; Barrière et al., 2008; Torres et al., 2015), alfalfa (Sheaffer et al., 1998) and switchgrass (Hopkins et al., 1995), the variation attributed to the genotype-by-environment interaction effect is considerably smaller than the variation attributed to the genotype and environment main effects (Tables 4, 5).

To further examine accession differences in environmental sensitivity, accession performance across locations was studied in more detail using the data from the third harvest year (Table 7). The largest variation in cellulose content across locations was observed for OPM 12, while the largest variation in contents of hemicellulosic polysaccharides and lignin was observed for OPM 2. Similarly, OPM 9 displayed the largest variation for NDF and CEY, while OPM 10 and OPM 3, respectively displayed the largest variation in CelCon and HemCon.

To study such differences in the stability of accession performance, a Finlay Wilkinson stability analysis (Finlay and Wilkinson, 1963) was performed on CEY data of the third cultivation year, to estimate the environmental sensitivity of accessions for this trait (Table 8). The higher the sensitivity estimate, the more sensitive an accession is to the “quality” of the growth location for the evaluated trait. The environmental quality in this analysis refers to deviation of mean accession performance in that location from the mean accession performance over all evaluated locations. Accession performance of OPM 1 was found to be the least sensitive (sensitivity 0.54) and OPM 9 the most sensitive (sensitivity 1.50) to environmental quality (Table 8). The static stability parameter of each accession was also calculated, which is a measure of the variance in accession performance across locations (Becker and Leon, 1988). A smaller static stability means smaller variation in accession performance across locations. Accession performance of OPM 1 was the most stable (static stability 30) and OPM 10 the least stable (static stability 119) across environments (Table 8). The superiority coefficient is used to identify accessions that perform relatively well in all test locations and accounts for both mean performance and stability (Lin and Binns, 1988). OPM 6 ranked first in overall performance across environments (lowest superiority coefficient), while OPM 9 ranked last (Table 8).

**Table 8:** **Environmental sensitivity and genotype stability and superiority scores for calculated ethanol yield (g/kg dm) of 15 miscanthus accessions evaluated across six locations (cultivation year 3, 2014–2015)**.

**Accession**	**Mean CEY**	Environmental sensitivity^*^	**Static stability^¥^**	**Superiority coefficient^§^**	**Superiority rank^‡^**
OPM 1	73.05	0.54	29.65	132.10	11
OPM 2	84.05	0.66	52.48	17.40	2
OPM 3	71.51	0.93	55.73	161.90	13
OPM 4	79.53	1.13	76.25	54.10	7
OPM 5	75.23	0.93	43.31	103.70	9
OPM 6	86.64	0.78	34.05	16.30	1
OPM 7	71.13	1.21	65.50	174.00	14
OPM 8	73.13	0.99	49.76	145.50	12
OPM 9	61.29	1.50	93.61	405.00	15
OPM 10	75.43	1.48	118.93	131.50	10
OPM 11	79.25	0.94	38.76	56.00	8
OPM 12	80.03	1.03	53.48	45.10	6
OPM 13	83.25	0.92	40.40	22.00	3
OPM 14	82.52	0.85	40.66	28.70	4
OPM 15	82.24	1.04	50.60	34.80	5

* Environmental sensitivity, the slope of the regression line of the fitted Finlay Wilkinson (FW) model;

¥ Static stability, the variance around the accession mean across environments;

§ Superiority coefficient, the mean square distance between accession performance and maximum observed performance in each environment;

‡ *Superiority rank, Accession ranking based on superiority coefficient*.

**Figure 4:**
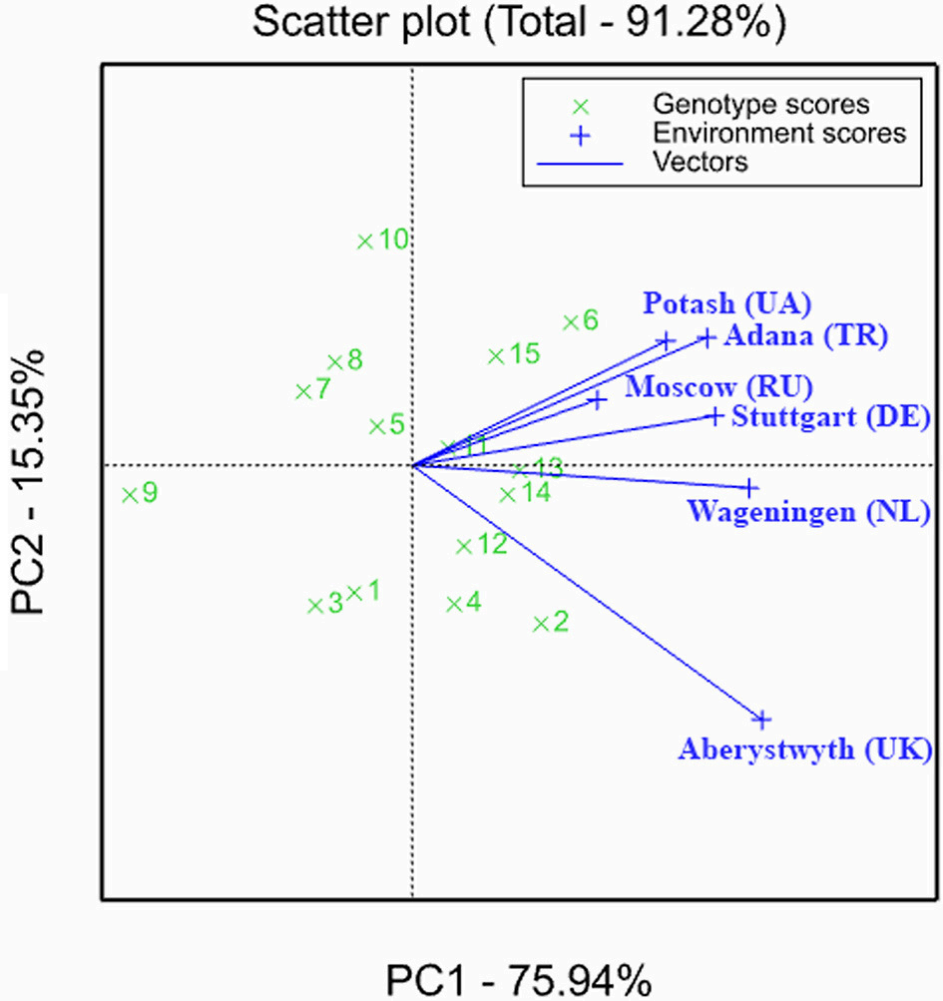
**GGL biplot of variation in accession performance in calculated ethanol yield (g/kg dm) across six locations in the third cultivation year (2014–2015). Numbers represent accession OPM codes**.

A useful tool to visualize the variation in accession performance across locations is the GGE biplot (Figure 4) (Yan et al., 2000; Yan and Kang, 2002; Malosetti et al., 2013). The origin of the plot represents the average performance of accessions across the environments, the length of environment vectors is proportional to the genetic variance within environments (the extent of variation among accessions within one environment) and the angle between vectors is proportional to the correlation between environments (Yan and Kang, 2002; Malosetti et al., 2013). The first two principal components visualized in the biplot explained 91.28% of the variation (Figure 3). The angle between the vector for Potash and the vector for Aberystwyth is almost 90 degrees, indicating that there is virtually no correlation in accession performance between these two locations. The perpendicular projection of accessions on the environment vectors approximates accession performance per environment, showing that OPM 2 performed the best in Aberystwyth, while OPM 6 performed the best in all other trial locations. OPM 9 performed the worst in all locations. Along with the previous observation that OPM-6 had the lowest superiority coefficient and the highest mean performance in terms of CEY across locations (Table 8), this shows that the calculated ethanol yield of OPM-6 was relatively insensitive to differences between locations and was superior to the other accessions in 5 out of 6 trial locations. The selection of stable accessions to counter the effects of genotype-by-location interactions is a viable approach if, like is the case here, the performance of the stable accession is not much lower compared to adapted accessions. However, the stable and superior accession OPM-6 did perform relatively poor in Aberystwyth compared to OPM-2, but still had average performance among all accessions.

### Implications for the use of miscanthus as a lignocellulose feedstock

There is a need for the development of novel miscanthus varieties with improved biomass quality for processing into cellulosic ethanol and other bioproducts. The large extent of observed genotypic variation in cell wall composition and saccharification efficiency observed in this study indicates potential for the selection of miscanthus accessions with favorable biomass quality characteristics. However, in addition to genetic factors also environmental factors substantially affect cell wall composition and conversion efficiency. This can be highly problematic, as a consistent supply of biomass of predictable composition and high quality is a crucial factor for the success of lignocellulose biorefineries (Perlack et al., 2005). Also from a breeding perspective a large environmental influence on the trait of interest is undesirable, as the environmentally derived part of the phenotypic variation is hard to control. This is especially problematic if the effect is unpredictable due to unknown and/or fluctuating environmental stimuli.

To combat this, genotypes with a stable and good performance across diverse locations are ideal. Differences in environmental sensitivity among the tested accessions were evident. However, accession ranking also varied across locations, which implicates that an accession that performs well in one location may not perform well in another. Stability analysis of accession performance for CEY in the third cultivation year, identified OPM-6 as a stable and superior accession, which had the best performance in 5 out of 6 locations and average performance in the remaining trial location. The selection of genotypes with a stable and superior performance across environments may thus be a viable approach, but it requires that breeding germplasm is evaluated in multiple and diverse locations.

Trials also need to be conducted over multiple years, before selections can be made reliably. Miscanthus is a perennial crop that matures in approximately 3 years and accession performance differed substantially between cultivation years. It was observed that establishment rates of miscanthus varied between the locations, with faster establishment of miscanthus in Adana than in the other locations. However, in all evaluated locations, accession performance for CEY in the second cultivation year was predictive of that at full maturity with reasonable accuracy, indicating that selections can be reliably made from the second cultivation year onwards.

The obtained results highlight the potential impact of environmental conditions and cultivation year on the quality of miscanthus biomass for biofuel production, but - in a wider perspective - are also relevant to many other potential biomass value-chains. Especially processes that rely on biomass fractionation, such as refinery processes, whose techno-economic efficiency may be considerably affected by such variation in cell wall. To increase our understanding of which environmental stimuli are the cause of the observed environmentally derived variation cell wall composition and conversion efficiency, further research is needed in which a broader range of environments is evaluated. In this way the most suitable production environment can be identified given certain biomass quality criteria posed by the end-user. Simultaneously, selection for biomass quality in miscanthus through breeding should take into account these effects of environmental factors and cultivation year on accession performance in order to identify stable and superior genotypes that consistently yield high quality biomass across diverse production environments. The influence of environmental conditions on biomass quality is substantial and should be taken into account in order to match genotype, location and end-use of miscanthus as a lignocellulose feedstock.

## Author contributions

LT, OD, and TV: Designed and planned the experiments; TV: Perform the experiments; TV: Wrote the first draft and LT, OD, and RV revised the manuscript.

### Conflict of interest statement

The authors declare that the research was conducted in the absence of any commercial or financial relationships that could be construed as a potential conflict of interest.
