# Supplementary material for: Stability of Cell Wall Composition and Saccharification Efficiency in Miscanthus across Diverse Environments
Source: Front Plant Sci. 2017 Jan 5;7:2004. doi: 10.3389/fpls.2016.02004 (PMC5216675; doi:10.3389/fpls.2016.02004)
Supplement: Supplementary file 1 [file DataSheet1.DOCX]

***Supplementary Table S1***. *Analyses of variance for cell wall composition of 15 miscanthus genotypes grown in six locations (cultivation year 3, 2014-2015)*

| **Source of variation^*^** | **Degrees of freedom** | **NDF (g/kg dm)** | | **CEL (g/kg dm)** | | **HEM (g/kg dm)** | | **LIG (g/kg dm)** | |
| --- | --- | --- | --- | --- | --- | --- | --- | --- | --- |
|  |  | *Mean squares* | *F prob.* | *Mean squares* | *F prob.* | *Mean squares* | *F prob.* | *Mean squares* | *F prob.* |
| L | 5 | 37557.1 | <.001 | 46260.2 | <.001 | 16794.5 | <.001 | 3627.53 | <.001 |
| Residual^1^ | 12 | 504.1 |  | 802.4 |  | 572.2 |  | 122.31 |  |
| G | 14 | 3797.6 | <.001 | 8102.4 | <.001 | 15565.7 | <.001 | 2755.26 | <.001 |
| GL | 70 | 907.8 | <.001 | 896.9 | <.001 | 388 | <.001 | 134.73 | <.001 |
| Residual^2^ | 162 | 226.1 |  | 276.7 |  | 145 |  | 48.99 |  |

*^*^G = Genotype, L = Location, Y = Year, GL = Genotype-by-location interaction, GY = Genotype-by-year interaction, LY = Location-by-year interaction, GLY = Genotype-by-location-by-year interaction, Residual^1^ = Residual block stratum, Residual^2^ = Residual block*units stratum*

***Supplementary Table S2***. *Analyses of variance for conversion efficiency characters and calculated ethanol yield (CEY) of 15 miscanthus genotypes grown in six locations (cultivation year 3, 2014-2015)*

| **Source of variation** | **Degrees of freedom** | **CelCon (%)** | | **HemCon (%)** | | **CEY (g/kg dm)** | |
| --- | --- | --- | --- | --- | --- | --- | --- |
|  |  | *Mean squares* | *F prob.* | *Mean squares* | *F prob.* | *Mean squares* | *F prob.* |
| L | 5 | 427.911 | <.001 | 66.497 | <.001 | 1743.55 | <.001 |
| Residual^1^ | 12 | 9.022 |  | 1.757 |  | 23.85 |  |
| G | 14 | 126.924 | <.001 | 17.211 | <.001 | 777.82 | <.001 |
| GL | 70 | 11.221 | <.001 | 1.722 | 0.011 | 56.17 | <.001 |
| Residual^2^ | 162 | 3.853 |  | 1.099 |  | 23.89 |  |

*^*^G = Genotype, L = Location, Y = Year, GL = Genotype-by-location interaction, GY = Genotype-by-year interaction, LY = Location-by-year interaction, GLY = Genotype-by-location-by-year interaction, Residual^1^ = Residual block stratum, Residual^2^ = Residual block*units stratum*
